# Three-graded stratification of carbohydrate restriction by level of baseline hemoglobin A1c for type 2 diabetes patients with a moderate low-carbohydrate diet

**DOI:** 10.1186/1743-7075-11-33

**Published:** 2014-07-28

**Authors:** Hajime Haimoto, Tae Sasakabe, Takahiko Kawamura, Hiroyuki Umegaki, Masashi Komeda, Kenji Wakai

**Affiliations:** 1 Department of Internal Medicine, Haimoto Clinic, 1-80 Yayoi, Kasugai, Aichi 486-0838, Japan; 2 Department of Clinical Nutrition, Haimoto Clinic, 1-80 Yayoi-cho, Kasugai, Aichi 486-0838, Japan; 3 Department of Diabetes and Endocrine Internal Medicine, Chubu Rosai Hospital, 10-6-1, Komei-cho, Minato-ku, Nagoya, Aichi 455-8530, Japan; 4 Department of Geriatrics, Nagoya University Graduate School of Medicine, 65 Tsuruma-cho, Showa-ku, Nagoya, Aichi 466-8550, Japan; 5 Department of Cardiac Surgery, Kansai Heart Center, Nara 1-3-3 Ukyo Nara-city, 631-0805, Japan; 6 Department of Preventive Medicine, Nagoya University Graduate School of Medicine, 65 Tsuruma-cho, Showa-ku, Nagoya, Aichi 466-8550, Japan

**Keywords:** Low-carbohydrate diet, Carbohydrate intake, Macronutrient, Hemoglobin A1c, Stratification, Type 2 diabetes

## Abstract

**Background:**

A moderate low-carbohydrate diet has been receiving attention in the dietary management of type 2 diabetes (T2DM). A fundamental issue has still to be addressed; how much carbohydrate delta-reduction (Δcarbohydrate) from baseline would be necessary to achieve a certain decrease in hemoglobin A1c (HbA1c) levels.

**Objective:**

We investigated the effects of *three*-graded stratification of carbohydrate restriction by patient baseline HbA1c levels on glycemic control and effects of Δcarbohydrate on decreases in HbA1c levels (ΔHbA1c) in each group.

**Research design and methods:**

We treated 122 outpatients with T2DM by *three*-graded carbohydrate restriction according to baseline HbA1c levels (≤ 7.4% for Group 1, 7.5%-8.9% for Group 2 and ≥ 9.0% for Group 3) and assessed their HbA1c levels, doses of anti-diabetic drugs and macronutrient intakes over 6 months.

**Results:**

At baseline, the mean HbA1c level and carbohydrate intake were 6.9 ± 0.4% and 252 ± 59 g/day for Group 1 (n = 55), 8.1 ± 0.4% and 282 ± 85 g/day for Group 2 (n = 41) and 10.6 ± 1.4% and 309 ± 88 g/day for Group 3 (n = 26). Following *three*-graded carbohydrate restriction for 6 months significantly decreased mean carbohydrate intake (g/day) and HbA1c levels for all patients, from 274 ± 78 to 168 ± 52 g and from 8.1 ± 1.6 to 7.1 ± 0.9% (n = 122, *P* < 0.001 for both) and anti-diabetic drugs could be tapered. ΔHbA1c and Δcarbohydrate were -0.4 ± 0.4% and -74 ± 69 g/day for Group 1, -0.6 ± 0.9% and -117 ± 78 g/day for Group 2 and -3.1 ± 1.4% and -156 ± 74 g/day for Group 3. Linear regression analysis showed that the greater the carbohydrate intake, the greater the HbA1c levels at baseline (*P* = 0.001). Also, the greater the reduction in carbohydrate intake (g/day), the greater the decrease in HbA1c levels (*P* < 0.001), but ΔHbA1c was not significantly influenced by changes in other macronutrient intakes (g/day).

**Conclusions:**

Three-graded stratification of carbohydrate restriction according to baseline HbA1c levels may provide T2DM patients with optimal objectives for carbohydrate restriction and prevent restriction from being unnecessarily strict.

## Introduction

A low-carbohydrate diet (LCD) is defined as strict carbohydrate restriction to less than 130 g/day or less than 30% carbohydrate [1,2]. LCDs have beneficial effects on glycemic control, weight loss and serum lipid profiles compared to high-carbohydrate low-fat (energy-restricted) diets [2-4]. Although long-term safety has not been proved by interventional studies, no serious harm has resulted from following LCDs for several years [2,5-7].

A moderate LCD is defined as modest carbohydrate restriction to more than 130 g/day or 30 - 45% carbohydrate [1,2]. Moderate LCDs may be sufficiently effective for glycemic control in Japanese patients with type 2 diabetes because the proportion of energy obtained from carbohydrates or fat in East Asian populations, including Japanese, is quite different from that in Western populations: higher carbohydrate (about 55 - 60%) and lower fat percentages (about 20 - 25%) in Japanese population [8], versus lower carbohydrate and higher fat percentages in American population [9]. Accordingly, we modified the LCD to suit Japanese patients with type 2 diabetes. The moderate LCD we have used has been shown to be effective in reducing hemoglobin A1c (HbA1c) levels in Japanese diabetic patients with lower to higher HbA1c levels without reinforcement with anti-diabetic drugs [6,10-12]. The principle of the moderate LCD in our previous studies was two-graded stratification of carbohydrate restriction according to the patient’s baseline HbA1c level (2-graded moderate LCD) [6,10,11]. Patients with HbA1c levels < 9.0% were instructed to follow a 40 - 45% carbohydrate diet, while those with HbA1c levels ≥ 9.0% were instructed to follow a 30 - 33% carbohydrate diet; the former patients achieved a HbA1c reduction of 0.7% in 1–2 years [6,11], while the latter achieved a remarkable reduction of 3.1 - 3.6% in 6–12 months [10,11].

With the above 2-graded moderate LCD, however, both type 2 diabetic patients with lower HbA1c levels and patients with moderate type 2 diabetes were included in one group and both were required to follow a 40 - 45% carbohydrate diet, though less aggressive carbohydrate restriction might have been effective enough for the patients with lower HbA1c levels. We believe that *three*-graded stratification (3-graded moderate LCD) is more applicable to patients with a wide range of baseline HbA1c levels, especially those with lower HbA1c levels.

There is another fundamental issue to consider in achieving proper control of type 2 diabetes with any type of LCD. In many previous LCDs, absolute goals for carbohydrate intake, such as 150 g/day, were pre-set before starting them [3,6,10-17], which ignored patients’ own baseline carbohydrate intakes. We believe that delta-reduction of carbohydrate can make the outcome more predictable than absolute values. In this study, therefore, we set a goal in terms of carbohydrate delta-reduction from baseline intake rather than an absolute goal.

Thus, we designed this study to investigate the effects of a 3-graded moderate LCD based on patient baseline HbA1c levels (3 levels as opposed to 2 previously) on glycemic control and also the effects of Δcarbohydrate (g/day) rather than absolute volume on ΔHbA1c in each group.

## Patients and methods

Between April 2010 and December 2011, we recruited all new outpatients with type 2 diabetes of Haimoto Clinic with HbA1c levels of 6.5% or above for this study. We excluded patients with serum creatinine levels greater than 2.0 mg/dl (176.8 μmol/l), ketoacidosis, soft drink ketosis [18], cancer or liver cirrhosis. Patients being treated with insulin were also excluded because combination therapy consisting of a moderate LCD and insulin has not been investigated. We also excluded patients who were following carbohydrate restriction at baseline based on commercial diet therapies, e.g. Atkins diet.

Of 138 eligible Japanese outpatients, 8 declined to participate, 6 were voluntarily lost to follow-up and 2 moved, and thus 122 patients (72 men and 50 women; age: mean ± SD: 60 ± 9 years, range: 34–77 years) were investigated. After obtaining written informed consent, patients were followed up for 6 months.

The main protocol for the present study was approved by the Ethical Committee of the Nagoya Tokushukai General Hospital (approval number: 2010-2-104) and it was also registered in University Hospital Medical Network (UMIN000003425) before its start. The analysis regarding an association between Δintake of carbohydrate and ΔHbA1c levels was also approved by the Ethical Committee.

### Carbohydrate-reduced diet

The main principle of our moderate LCD is to eliminate carbohydrate-rich food once or twice daily, at breakfast and/or dinner [6,10-12]. We have instructed patients to avoid carbohydrate-rich food in accordance with a list as reported previously [6]. For the present research, based on the results of our previous studies, we added a less strict carbohydrate restriction category for the type 2 diabetic patients with lower HbA1c levels so patients were divided into 3 groups according to their baseline HbA1c: ≤ 7.4% (Group 1), 7.5% - 8.9% (Group 2) and ≥ 9.0% (Group 3). Patients with HbA1c levels ≤ 7.4% were instructed to restrict carbohydrate-rich food to half of the usual amount at dinner, those with 7.5% - 8.9% were instructed to eliminate it at dinner and those with levels ≥ 9.0% to eliminate it at both dinner and breakfast. Patients were not required to calculate daily carbohydrate intakes. While patients were forbidden to consume carbohydrate-containing foods between meals, they were permitted to eat as much protein and fat as they wished, including saturated fats. There were no other restrictions.

An experienced dietician (Tae Sasakabe) performed all the dietary assessments and gave instructions to all the participants. The patients had not followed LCDs before the present intervention. At baseline and after 6 months, patients’ dietary intakes were assessed based on 3-day dietary records. They were requested to maintain their usual level of physical activity throughout the study.

### Clinical assessment

We measured the body mass index (BMI), blood pressure (BP) and HbA1c level of each patient every month. Venous blood samples were obtained after an overnight (12-h) fast at baseline and 6 months for the determination of fasting plasma glucose (FPG), fasting serum insulin (IRI), triglyceride, LDL cholesterol, HDL cholesterol and creatinine levels.

We also recorded the doses of lipid-lowering or anti-diabetic drugs (glibenclamide, gliclazide, glimepiride, nateglinide, metformin, pioglitazone, voglibose, sitagliptin) taken by the patients.

### Laboratory methods

The HbA1c levels were measured by high-performance liquid chromatography (Arkley Co., Kyoto, Japan) and estimated as National Glycohemoglobin Standardization Program (NGSP) values (%) calculated by the formula HbA1c (%) = HbA1c (Japan Diabetes Society, JDS) (%) × 1.02 + 0.25 [19].

Plasma glucose concentrations were determined using enzymatic methods (Shino-Test Co., Kanagawa, Japan). Serum insulin levels were measured using the standard double antibody radioimmunoassay method (Fujirebio Inc., Tokyo, Japan). Enzymatic methods were used to measure serum creatinine and triglyceride concentrations (Daiichi Pure Chemicals Co., Tokyo, Japan). Direct methods were used to assay serum LDL cholesterol and HDL cholesterol levels (Daiichi Pure Chemicals Co., Tokyo, Japan).

### Statistical analysis

The parameter change for each biomarker (Δ) was defined as the level after 6 months minus the level at baseline.

The Wilcoxon test was used to assess the changes in HbA1c levels and other cardiovascular risk factors, total energy and macronutrient intakes due to our moderate LCD, and compare the levels between baseline and after 6 months for all patients. We computed Spearman’s correlation coefficients (r_s_) to examine correlations between Δcabohydrate (g/day), Δ%carbohydrate (proportion of energy from carbohydrate in total energy intake) or Δother macronutrients and ΔHbA1c. Multiple regression analyses were performed to examine associations between Δcabohydrate (g/day) or Δ%carbohydrate and ΔHbA1c with adjustment for changes in energy intake (Δtotal energy intake).

Increasing or decreasing trends in characteristics with increasing baseline HbA1c levels were tested by linear regression models including a score of 1, 2 and 3 given to Group1, Group 2 and Group 3, respectively. Total energy intake was adjusted using the regression model. We indicated changes in clinical (body mass index), biological (HbA1c, FPG, IRI and serum lipid profiles) and nutritional [total energy intake and macronutrient intake (g/day and %energy)] variables separately for each of the 3 groups. Then, increasing or decreasing trends in these changes with increasing baseline HbA1c levels were tested by linear regression models including the scores mentioned above. The total energy intake or Δtotal energy intake was adjusted using linear regression.

In addition, increasing or decreasing trends in ΔHbA1c with increasing Δtotal energy intake were tested by linear regression models including a score of 1, 2 and 3 given to Δtotal energy Group 1, 2 and 3 stratified by tertile of Δtotal energy intake, respectively. Furthermore, patients with > 80% decreases in total energy intake due to reductions in carbohydrate intake were defined as carbohydrate reduction predominant patients and others as carbohydrate reduction less predominant patients. In each group, we compared Δtotal energy and ΔHbA1c between carbohydrate reduction predominant and less predominant patients using the *t*-test or Mann–Whitney test as appropriate.

*P*-values less than 0.05 were considered statistically significant. Data are shown as mean ± SD. All statistical analyses were performed using SPSS (version 15.0; SPSS, Inc., Chicago, IL, USA).

## Results

### Baseline characteristics of patients according to their baseline HbA1c levels

The baseline characteristics of the 122 patients including clinical and biological parameters are shown in Table 1. The mean HbA1c level was 8.1 ± 1.6% (range: 6.5 - 14.1%). The mean levels were 6.9 ± 0.4% for Group 1, 8.1 ± 0.4% for Group 2 and 10.6 ± 1.4% for Group 3.

**Table 1:** Baseline characteristics of patients according to baseline HbA1c levels, and changes in HbA1c levels and cardiovascular risk factors during 6 months (n = 122)

	**All patients**		Baseline hemoglobin A1c (%)*				
			**Group 1 ≤ 7.4%**	**Group 2 7.5 - 8.9%**	**Group 3 ≥ 9.0%**	***P *** **for trend**	**Energy-adjusted *P *** **for trend**
	**n = 122**		**n = 55**	**n = 41**	**n = 26**		
Baseline							
Male/female (n)	72/50		30/25	27/14	15/11		
Age (years)	60.4 ± 9.3		62.2 ± 7.7	60.2 ± 10.1	56.6 ± 10.1	0.011	0.033
Duration of diabetes (months)	47 ± 66		32.1 ± 57.6	66.9 ± 75.2	47.8 ± 62.1	0.139	0.044
Hemoglobin A1c (%)	8.1 ± 1.6		6.9 ± 0.4	8.1 ± 0.4	10.6 ± 1.4	< 0.001	< 0.001
Fasting plasma glucose (mmol/l)	8.10 ± 2.39		6.66 ± 0.61	8.27 ± 1.66	10.8 ± 3.1	< 0.001	< 0.001
Fasting serum insulin (pmol/l)	44.3 ± 25.9		44.5 ± 26.9	44.6 ± 25.9	43.8 ± 24.4	0.918	0.583
Body mass index	24.8 ± 3.9		23.9 ± 3.8	25.6 ± 4.3	25.3 ± 2.8	0.061	0.205
Serum triglyceride (mmol/l)	1.50 ± 1.18		1.39 ± 1.02	1.72 ± 1.57	1.40 ± 0.62	0.721	0.886
Serum HDL-cholesterol (mmol/l)	1.45 ± 0.39		1.50 ± 0.39	1.37 ± 0.39	1.47 ± 0.31	0.653	0.877
Serum LDL-cholesterol (mmol/l)	3.39 ± 0.85		3.31 ± 0.83	3.36 ± 0.91	3.65 ± 0.80	0.112	0.073
Changes during 6 months		*P*†					
Δhemoglobin A1c (%)*	-1.0 ± 1.4	< 0.001	-0.4 ± 0.4	-0.6 ± 0.9	-3.1 ± 1.4	< 0.001	< 0.001
Δfasting plasma glucose (mmol/l)	-0.83 ± 1.94	< 0.001	-0.33 ± 1.05	-0.33 ± 1.89	-2.89 ± 2.16	< 0.001	< 0.001
Δserum insulin (pmol/l)	-4.08 ± 19.2	0.010	4.92 ± 21.8	-6.06 ± 15.7	1.14 ± 16.5	0.05	0.220
Δbody mass index	-0.86 ± 1.20	< 0.001	-0.81 ± 1.14	-0.91 ± 1.26	-0.86 ± 1.29	0.803	0.978
Δserum triglyceride (mmol/l)	-0.18 ± 0.95	0.020	-0.06 ± 0.77	-0.31 ± 1.28	-0.21 ± 0.67	0.365	0.331
Δserum HDL-cholesterol (mmol/l)	0.10 ± 0.26	< 0.001	0.05 ± 0.28	0.10 ± 0.23	0.18 ± 0.23	0.022	0.024
Δserum LDL-cholesterol (mmol/l)	-0.18 ± 0.85	0.004	-0.08 ± 0.80	-0.18 ± 0.83	-0.39 ± 1.01	0.156	0.309

Data are shown as mean ± SD. The participants were divided into three groups according to baseline hemoglobin A1c.

*Hemoglobin A1c (mmol/mol) = 10.93 × National Glycohemoglobin Standardization Program (%) - 23.52. *P*†: Wilcoxon test between baseline and after 6 months in all patients.

### Correlations of HbA1c levels with macronutrients at baseline

**Figure 1:**
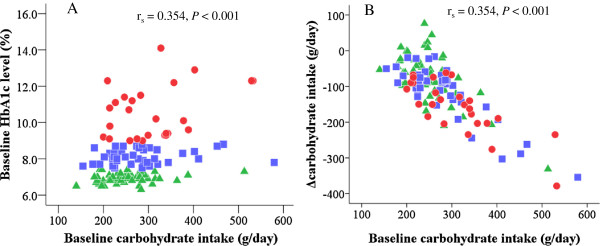
**Correlation of baseline HbA1c levels with baseline carbohydrate intake and that of Δcarbohydrate intake with baseline carbohydrate intake.** Green triangles indicate patients with HbA1c levels of ≤ 7.4%, blue squares those with HbA1c levels of 7.5% - 8.9% and red circles those with HbA1c levels of ≥ 9.0%. Baseline HbA1c levels were positively correlated with baseline carbohydrate intake (g/day) **(A)**. Δcarbohydrate intake (g/day) was inversely and very strongly correlated with baseline carbohydrate intake (g/day) with consumption of the moderate LCDs **(B)**.

Baseline HbA1c levels were positively correlated with baseline carbohydrate intake (g/day) (Figure 1A) and positively, though weakly, with total energy intake (r_s_ = 0.292, *P* = 0.001), but not with baseline %carbohydrate (r_s_ = 0.152, *P* = 0.095). The correlation of baseline HbA1c levels with baseline carbohydrate intake (g/day) remained significant even after adjustment for total energy intake in linear regression analysis (*P* = 0.015). Baseline HbA1c levels were not significantly correlated with baseline fat intake, fat percentage in total energy intake (%fat) and protein intake, but there was a weak, inverse correlation with baseline protein percentage in total energy intake (%protein) (r_s_ = -0.250, *P* = 0.004).

We observed significant increasing trends in total energy and carbohydrate intakes (g/day) and a decreasing trend in %protein with an increasing trend in baseline HbA1c levels, but this trend in baseline carbohydrate intake (g/day) was no longer significant after adjustment for total energy intake (Table 2). No significant trend was found for %carbohydrate or other indices of macronutrients with increasing baseline HbA1c levels.

**Table 2:** Baseline intakes of total energy and macronutrients and their changes during 6 months by baseline hemoglobin A1c levels (n = 122)

**Energy and nutrients**	**All patients**		Baseline hemoglobin A1c (%)*				
			**Group 1 ≤ 7.4%**	**Group 2 7.5 - 8.9%**	**Group 3 ≥ 9.0%**	***P *** **for trend**	**Energy-adjusted *P *** **for trend**
	**n = 122**		**n = 55**	**n = 41**	**n = 26**		
Baseline							
Total energy intake (kcal/day)	2047 ± 549		1914 ± 381	2048 ± 595	2323 ± 678	0.002	
Carbohydrate intake (g/day)	274 ± 78		252 ± 59	282 ± 85	309 ± 88	0.001	0.209
%carbohydrate (%)	54.0 ± 8.2		52.9 ± 7.8	55.6 ± 8.3	54.1 ± 9.0	0.349	0.088
Fat intake (g/day)	58 ± 20		57.2 ± 17.9	53.7 ± 19.4	65.1 ± 23.2	0.206	0.177
%fat (%)	25.4 ± 6.1		26.8 ± 6.6	23.5 ± 5.1	25.3 ± 5.9	0.130	0.126
Protein intake (g/day)	77 ± 22		75.0 ± 19.1	77.8 ± 21.6	80.7 ± 26.8	0.259	0.073
%protein (%)	15.3 ± 2.7		15.8 ± 2.6	15.5 ± 3.0	13.9 ± 2.1	0.007	0.047
Changes during 6 months		*P*†					
Δtotal energy intake (kcal day)	-399 ± 508	< 0.001	-323 ± 430	-338 ± 500	-657 ± 601	0.012	
Δcarbohydrate intake (g/day)	-106 ± 80	< 0.001	-74 ± 69	-117 ± 78	-156 ± 74	< 0.001	< 0.001
Δ%carbohydrate (%)	-12.5 ± 10.0	< 0.001	-8.0 ± 9.2	-15.6 ± 8.5	-16.9 ± 10.4	< 0.001	< 0.001
Δfat intake (g/day)	6 ± 27	0.041	0.7 ± 24.6	16.3 ± 27.9	2.0 ± 24.5	0.394	0.002
Δ%fat (%)	9.1 ± 9.3	< 0.001	5.5 ± 8.9	12.6 ± 9.0	11.4 ± 8.1	0.001	0.051
Δprotein intake (g/day)	-0.4 ± 22.8	0.938	-1.5 ± 20.8	1.3 ± 20.9	-1.0 ± 29.8	0.826	0.011
Δ%protein (%)	3.6 ± 3.8	< 0.001	2.9 ± 3.4	3.3 ± 3.9	5.4 ± 3.8	0.009	0.036

Data are shown as mean ± SD. The participants were divided into 3 groups according to baseline hemoglobin A1c levels. The parameter change for each biomarker (Δ) was defined as the level after 6 months minus the level at baseline. *Hemoglobin A1c (mmol/mol) = 10.93 × National Glycohemoglobin Standardization Program (%) - 23.52. *P*†: Wilcoxon test between baseline and after 6 months in all patients.

### Changes in macronutrients during 6 months

The daily carbohydrate intake ranged widely at baseline, from 140 g to 579 g. The average carbohydrate intake (g/day) significantly decreased, from 274 ± 78 g (54 ± 8% of total energy) at baseline to 168 ± 52 g (41 ± 11%) after 6 months (Table 2). The total energy intake also significantly decreased. There was a slight increase in fat intake but it was significant. While there was no change in protein intake over 6 months, %protein significantly increased due to a marked decline in total energy intake.

At the end of the study, intakes of total energy and carbohydrate were 1591 ± 361 kcal/day and 178 ± 54 g/day (44.8 ± 9.9%) for Group 1, 1710 ± 424 kcal/day and 165 ± 44 g/day (39.9 ± 10.8%) for Group 2 and 1666 ± 455 kcal/day and 153 ± 54 g/day (37.1 ± 10.2%) for Group 3. Intakes of fat and protein were 58 ± 22 g/day (32.2 ± 7.7%) and 74 ± 18 g/day (18.7 ± 3.1%) for Group 1, 70 ± 30 g/day (36.1 ± 9.3%) and 80 ± 24 g/day (19.4 ± 3.3%) for Group 2 and 67 ± 20 g/day (36.8 ± 8.1%) and 80 ± 24 g/day (19.4 ± 3.3%) for Group 3, respectively.

Δcarbohydrate (g/day) was inversely and very strongly correlated with baseline carbohydrate intake (g/day) (Figure 1B), while Δ%carbohydrate was inversely and weakly correlated with baseline %carbohydrate (r_s_ = -0.226, *P* = 0.012) as a result of following our moderate LCD for 6 months.

### Changes in HbA1c levels and other cardiovascular risk factors during 6 months in all patients

Compared to baseline, the mean HbA1c and FPG levels significantly decreased over 6 months, from 8.1 ± 1.6 to 7.1 ± 0.9% and 8.10 ± 2.39 to 7.21 ± 1.55 mmol/l (*P* < 0.001 for both). The mean IRI levels and BMI also significantly decreased, from 44.3 ± 25.9 to 40.3 ± 25.8 pmol/l and 24.8 ± 3.9 to 23.9 ± 3.7 (*P* = 0.010 for IRI and *P* < 0.001 for BMI) (Table 1).

Serum lipid profiles, including serum triglyceride, HDL-cholesterol and LDL-cholesterol, significantly improved in all patients as a result of following our moderate LCD for 6 months (Table 1). After 39 patients taking lipid-lowering drugs were excluded (n = 83), there were still significant improvements in serum HDL-cholesterol (*P* < 0.001) and LDL-cholesterol (*P* = 0.021). While there was also a decrease in serum triglyceride, it was not statistically significant (*P* = 0.120).

### Correlation of changes in HbA1c levels with those in carbohydrate intake during 6 months in all patients

**Figure 2:**
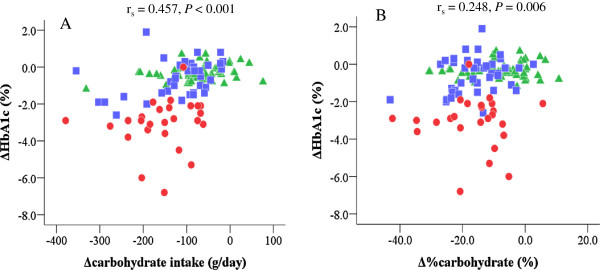
**Correlations of ΔHbA1c with Δcarbohydrate.** Green triangles indicate patients with HbA1c levels of ≤ 7.4%, blue squares those with HbA1c levels of 7.5% - 8.9% and red circles those with HbA1c levels of ≥ 9.0%. For all patients, ΔHbA1c was positively correlated with Δcarbohydrate (g/day) **(A)** and Δ%carbohydrate **(B)**. Focusing on individual Groups, the correlation of ΔHbA1c with Δcarbohydrate (g/day) was weaker in Group 1 (r_s_ = 0.281, *P* = 0.038), while stronger in Group 2 (r_s_ = 0.376, *P* = 0.015) and Group 3 (r_s_ = 0.349, *P* = 0.081).

ΔHbA1c was positively correlated with Δcarbohydrate intake (g/day) (r_s_ = 0.457, *P* < 0.001) (Figure 2A), Δ%carbohydrate (r_s_ = 0.248, *P* = 0.006) (Figure 2B) and Δtotal energy intake (r_s_ = 0.306, *P* = 0.001). However, focusing on the results for each group, the correlation of ΔHbA1c with Δcarbohydrate intake (g/day) was weaker in Group 1 (r_s_ = 0.281, *P* = 0.038), but stronger in Group 2 (r_s_ = 0.376, *P* = 0.015) and Group 3 (r_s_ = 0.349, *P* = 0.081).

ΔHbA1c was weakly and negatively correlated with Δ%protein (r_s_ = -0.253, *P* = 0.005). ΔHbA1c was not significantly correlated with Δprotein (g/day) (r_s_ = 0.086, *P* = 0.345), Δfat (g/day) (r_s_ = 0.038, *P* = 0.675) or Δ%fat (r_s_ = -0.157, *P* = 0.084).

In multiple regression analyses with adjustment for Δtotal energy intake, the correlations of Δcarbohydrate (g/day) or Δ%carbohydrate with ΔHbA1c remained significant (*P* = 0.002 for Δcarbohydrate [g/day] and *P* < 0.001 for Δ%carbohydrate). In contrast, the significant correlation of Δtotal energy intake with ΔHbA1c disappeared with adjustment for Δcarbohydrate (g/day) (*P* = 0.945), while a correlation remained with adjustment for Δ%carbohydrate (P < 0.001).

### Correlations of changes in HbA1c levels with those in BMI or serum lipid profiles during 6 months in all patients

**Figure 3:**
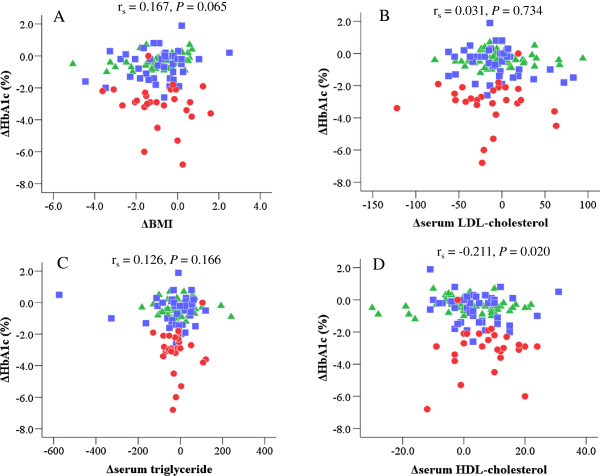
**Correlations of changes in HbA1c levels with those in BMI and serum lipid profiles during 6 months in all patients.** Green triangles indicate patients with HbA1c levels of ≤ 7.4%, blue squares those with HbA1c levels of 7.5% - 8.9% and red circles those with HbA1c levels of ≥ 9.0%. We found no correlation between ΔHbA1c and ΔBMI **(A)**, Δserum LDL-cholesterol **(B)** or Δserum triglyceride **(C)**, while there was a negative and weak correlation between ΔHbA1c and Δserum HDL-cholesterol **(D)**.

We found no correlation between ΔHbA1c and ΔBMI (r_s_ = 0.167, *P* = 0.065) (Figure 3A), Δserum LDL-cholesterol (r_s_ = 0.031, *P* = 0.734) (Figure 3B) or Δserum triglyceride (r_s_ = 0.126, *P* = 0.166, Figure 3C), while there was a negative and weak correlation between ΔHbA1c and Δserum HDL-cholesterol (r_s_ = -0.211, *P* = 0.020) (Figure 3D).

### Changes in HbA1c levels, cardiovascular risk factors and macronutrients across 3 groups

ΔHbA1c and Δcarbohydrate were -0.4 ± 0.4% and -74 ± 69 g/day for Group 1 (n = 55), -0.6 ± 0.9% and -117 ± 78 g/day for Group 2 (n = 41) and -3.1 ± 1.4% and -156 ± 74 g/day for Group 3 (n = 26), respectively (Tables 1 and 2). Decreasing trends in ΔHbA1c, ΔFPG, Δcarbohydrate intake and Δtotal energy intake were evident across the 3 groups. Regarding other macronutrients, significant increasing trends in Δ%fat and Δ%protein were also evident, but such trends for Δfat (g/day) and Δprotein (g/day) were not significant across the 3 groups. After adjustment for Δtotal energy intake, the trends remained materially the same except for those in Δfat intake and Δprotein intake, for which there was a significant increase after adjustment.

We found no correlations for changes in other cardiovascular risk factors, including Δbody mass index, ΔIRI, Δserum triglyceride and Δserum LDL-cholesterol, across the 3 groups, though an increasing trend in Δserum HDL-cholesterol was significant (Table 1). After adjustment for Δtotal energy intake, the significant trend remained. After excluding 39 patients who received lipid-lowering drugs, however, the trend in Δserum HDL-cholesterol across the 3 groups lost significance (*P* = 0.184).

In addition, trends in ΔHbA1c with increasing baseline IRI levels were tested by linear regression models including a score of 1, 2 and 3 given to tertile of baseline IRI levels: IRI-Group 1 (mean IRI level: 20.2 ± 5.22 pmol/l), IRI-Group 2 (38.9 ± 5.46 pmol/l) and IRI-Group 3 (74.4 ± 20.4 pmol/l), respectively. ΔHbA1c values were -0.8 ± 1.3% for IRI-Group 1, -1.3 ± 1.5% for IRI-Group 2 and -0.9 ± 1.4% for IRI-Group 3. We found no variation in ΔHbA1c across the three IRI Groups (*P* = 0.651).

### Comparison of ΔHA1c between carbohydrate reduction predominant and less predominant patients by tertile of Δtotal energy intake

**Figure 4:**
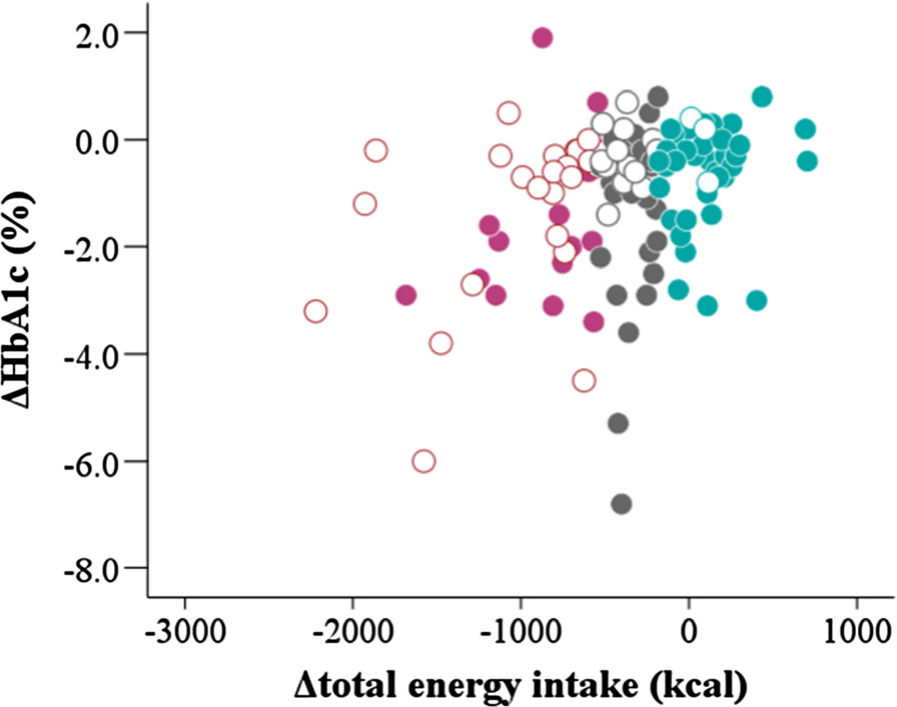
**Comparison of ΔHA1c between carbohydrate reduction predominant and less predominant patients in three Groups stratified by tertile of Δtotal energy intake.** Purple circles indicate Δtotal energy Group 1, gray circles Δtotal energy Group 2 and cobalt blue circles Δtotal energy Group 3. The mean decrease in HbA1c levels was greater in the patients with the highest reductions in carbohydrate intake (closed circles) than in those with lower reductions (open circles) in each Δtotal energy Group. The difference was statistically significant in Δtotal energy Group 2 (*p* = 0.008).

We analyzed data to examine whether the effect of Δcarbohydrate intake on ΔHbA1c was independent of Δtotal energy intake by comparison of ΔHb1c between carbohydrate reduction predominant and less predominant patients stratified by tertile of Δtotal energy intake (Δtotal energy Groups 1–3). The results are shown in Table 3 and Figure 4. The mean reduction in total energy intake was not significantly different between the carbohydrate reduction predominant patients and the carbohydrate reduction less predominant patients in the three Δtotal energy Groups. The decrease in HbA1c levels was greater in the patients with the highest reductions in carbohydrate intake than in those with lower reductions in each Δtotal energy Group. In particular, the difference was statistically significant in Δtotal energy Group 2 (*P* = 0.008).

**Table 3:** Comparison of ΔHA1c between carbohydrate reduction predominant and less predominant patients: stratified analysis by tertile of Δtotal energy intake

**Group**	**Δtotal energy (kcal/day)**	P*	**Energy from Δcarbohydrate (kcal/day)**	**Energy from Δfat (kcal/day)**	**ΔHbA1c† (%)**	***P***
Δtotal energy Group 1 (n = 41)	-939 ± 422					
Carbohydrate reduction predominant patients (n = 16)	-866 ± 325	0.557	-817 ± 264	-11 ± 140	-1.6 ± 1.5	0.185‡
Carbohydrate reduction less predominant patients (n = 25)	-984 ± 476		-591 ± 325	-165 ± 154	-1.3 ± 1.6	
Δtotal energy Group 2 (n = 41)	-347 ± 106					
Carbohydrate reduction predominant patients (n = 28)	-331 ± 104	0.178	-455 ± 211	113 ± 231	-1.4 ± 1.7	0.008§
Carbohydrate reduction less predominant patients (n = 13)	-380 ± 108		-214 ± 96	-82 ± 72	-0.3 ± 0.6	
Δtotal energy Group 3 (n = 40)	99 ± 208					
Carbohydrate reduction predominant patients (n = 37)	101 ± 216	0.980	-241 ± 184	256 ± 191	-0.6 ± 0.9	0.258‡
Carbohydrate reduction less predominant patients (n = 3)	74 ± 54		193 ± 104	-142 ± 44	-0.1 ± 0.6	

Data are shown as mean ± SD. P*: Mann–Whitney test between carbohydrate reduction predominant patients and less predominant patients.

†: Hemoglobin A1c (mmol/mol) = 10.93 × National Glycohemoglobin Standardization Program (%) - 23.52. ‡: Mann–Whitney test, §: *t*-test.

### Changes in anti-diabetic drugs

**Figure 5:**
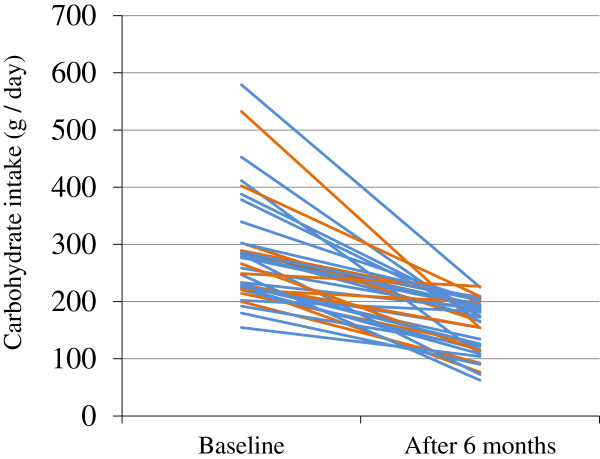
**Changes in carbohydrate intake (g/day) between patients with less medication and those with more medication over 6 months.** In 25 patients (blue lines), medication was eliminated or reduced in the study period while in 12 (orange lines), it was increased or newly started. The mean reduction in carbohydrate intake was greater in the former than in the latter, but the difference did not reach statistical significance (*P* = 0.095).

At baseline, 36 of the 122 patients (30%) had already been prescribed anti-diabetic drugs by other physicians (Table 4). At the end of the study, the number of patients taking anti-diabetic drugs had decreased to 17 (14%), half of the baseline number. In 25 patients, medication was eliminated or reduced in the study period while in 12, it was increased or newly started. The reduction in carbohydrate intake was greater in the former (-132 ± 86 g/day) than in the latter (-122 ± 86 g/day), but the difference did not reach statistical significance (*P* = 0.095) (Figure 5).

**Table 4:** Anti-diabetic drugs at baseline and after 6 months by baseline hemoglobin A1c levels (n = 122)

	**All patients**	Baseline hemoglobin A1c (%)*		
		**Group 1 ≤ 7.4%**	**Group 2 7.5 – 8.9%**	**Group 3 ≥ 9.0%**
	**n = 122**	**n = 55**	**n = 41**	**n = 26**
Baseline (n)	36 (30%)	6 (11%)	25 (61%)	5 (19%)
Glibenclamide	4 (3.45 mg)	0	3 (3.8 mg)	1 (2.5 mg)
Gliclazide	1 (120 mg)	0	1 (120 mg)	0
Glimepiride	22 (1.84 mg)	3 (1.5 mg)	15 (2.0 mg)	4 (1.5 mg)
Nateglinide	3 (120 mg)	0	3 (120 mg)	0
Metformin	12 (521 mg)	3 (417 mg)	8 (531 mg)	1 (750 mg)
Pioglitazone	9 (23 mg)	4 (23 mg)	2 (30 mg)	3 (20 mg)
Voglibose	16 (0.6 mg)	4 (0.7 mg)	10 (0.6 mg)	2 (0.9 mg)
Sitagliptin	8 (56 mg)	0	7 (57 mg)	1 (50 mg)
After 6 months (n)	17 (14%)	1 (2%)	12 (29%)	4 (15%)
Glibenclamide	2 (4.4 mg)	0	1 (7.5 mg)	1 (1.25 mg)
Gliclazide	1 (120 mg)	0	1 (120 mg)	0
Glimepiride	12 (1.4 mg)	1 (1.0 mg)	9 (1.6 mg)	2 (0.8 mg)
Nateglinide	0	0	0	0
Metformin	10 (600 mg)	0	7 (571 mg)	3 (667 mg)
Pioglitazone	1 (30 mg)	0	1 (30 mg)	0
Voglibose	5 (0.5 mg)	0	5 (0.5 mg)	0
Sitagliptin	4 (63 mg)	0	4 (63 mg)	0

The percentages indicate the proportion of patients that were prescribed each drug. Values in parentheses are the mean daily dose per person for anti-diabetic drugs. *Hemoglobin A1c (mmol/mol) = 10.93 × National Glycohemoglobin Standardization Program (%) - 23.52).

Twenty-five (61%) of the 41 patients in Group 2 were taking anti-diabetic drugs at baseline, but the number of patients on anti-diabetic drugs decreased from 25 to 12 during the 6 months, a more remarkable reduction than in the other 2 groups.

## Discussion

In the current study on Japanese patients with type 2 diabetes, a 3-graded moderate LCD with patients assigned to each grade according to baseline HbA1c (≤7.4%, 7.5% - 8.9% and ≥ 9.0%) led to remarkable reductions in both carbohydrate and total energy intakes. In spite of not having restrictions on total energy intake or fat intake, fat intake did not increase enough to compensate for the remarkable reduction in carbohydrate intake in this study. The remarkable decrease in total energy intake during 6 months, therefore, was almost certainly due to a great reduction in carbohydrate intake, not a reduction in fat intake. Under such circumstances, Δcarbohydrate (g/day) was correlated with ΔHbA1c independently of Δtotal energy intake [20].

Based on the results for the above nutritional changes, the 3-graded moderate LCD achieved good glycemic control - HbA1c levels of around 7.0% after 6 months in all groups despite the variation in baseline HbA1c levels - without reinforcement by anti-diabetic drugs.

The patients with lower HbA1c levels were assigned to Group 1 under the 3-graded moderate LCD and their carbohydrate intake was restricted the least severely. A -74 g-carbohydrate restriction in this group produced a -0.4% reduction in HbA1c after 6 months. If we had adopted the previous 2-graded moderate LCD, Group 1 would have been subjected to a -117 g-carbohydrate restriction. Thus, the carbohydrate restriction was lightened by 43 g in the patients with lower HbA1c levels. These results suggest that the 3-graded moderate LCD was effective for patients with lower HbA1c levels and avoided an unnecessarily strict carbohydrate restriction on them.

The diet was not as effective in patients in Group 2 as in the other groups. Reducing carbohydrate by 117 g decreased HbA1c by 0.6%. The effect of Δcarbohydrate on ΔHbA1c was smaller than expected. We assume one of the reasons to be that patients received less and less anti-diabetic drugs, especially sulfonylureas, in the course of the study because of concern about hypoglycemia. Thus, the effect of the 3-graded moderate LCD in Group 2 was actually better than it appeared from the results.

Awareness of hypoglycemia has recently increased because it is associated with a significant increase in macrovascular events, mortality and dementia [21,22] and hypoglycemia is a major adverse effect of anti-diabetic drugs, chiefly sulfonylureas [21]. Some patients did not restrict their carbohydrate intake as we instructed, while other patients over-restricted it. Therefore, the tapering of sulfonylurea doses in patients with lower HbA1c levels is a serious issue even with our moderate LCD. At the start of the moderate-LCD, we tapered the dose of sulfonylureas to about half of baseline to prevent hypoglycemia and monitored blood glucose levels every one or two weeks. Careful attention to dietary compliance and blood glucose levels is therefore necessary during the period from 1 to 2 months after beginning the moderate LCD.

We compared our results with two epoch-making studies reported by Westman et al. [14] and Gannon et al. [23]. First of all, American patients with type 2 diabetes had a lower carbohydrate intake (245 g/day) and greater BMI (30–38) than our Japanese subjects [14]. Regarding baseline HbA1c levels, Westman’s patients (mean HbA1c level: 8.3%) were close to our patients in Group 2 while Gannon’s patients (mean HA1c level: 10.0%) were close to our patients in Group 3. Westman’s patients achieved a 1.5% decrease in a HbA1c level corresponding to a 196 g/day reduction in carbohydrate intake, while the decrease in our Group 2 was much less. In view of this result, greater carbohydrate restriction should have been imposed on the patients in Group 2 in order to achieve a HbA1c level < 7.0% with tapering of anti-diabetic drugs. In contrast, a 30% carbohydrate diet (Δ%carbohydrate: -15%) led to a 2.5% reduction in HbA1c level in Gannon’s patients, while a 37% carbohydrate diet (Δ%carbohydrate: -17%) led to a 3.1% reduction in HbA1c levels in our patients in Group 3. This indicates that the 3-graded moderate LCD was sufficiently effective in our patients with a higher HbA1c level.

The 3-graded moderate LCD achieved similarly good results in patients in all groups. At the end of the study, daily carbohydrate intake and HbA1c levels were 153 g and 7.5% in Group 3, 165 g/day and 7.5% in Group 2 and 178 g and 6.4% in Group 1, respectively. The results were relatively close to each other in spite of the great difference in HbA1c levels and carbohydrate intakes at baseline, which ranged from 6.5 to 14.1% and 140 to 579 g/day at baseline, respectively. While patients with higher baseline HbA1c levels had been consuming larger amounts of carbohydrate, our moderate LCD regimen decreased carbohydrate intake to a greater extent in such patients. This suggests that we could adopt the diet for any baseline HbA1c level or amount of carbohydrate intake and achieve equally good results with respect to targets.

Deterioration of glycemic control in patients with higher HbA1c levels can be due to lower endogenous insulin secretion and/or poor dietary compliance. In the current study, ΔHbA1c was not associated with baseline IRI levels, though it was clearly correlated with Δcarbohydrate. Thus, a higher carbohydrate intake due to poor dietary compliance seems to be a more important as a cause of deterioration of glycemic control than endogeneous insulin secretion. However, this needs to be studied further because a correlation between Δinsulin secretion and Δcarbohydrate intake has still to be addressed.

Although it is ideal to calculate precise baseline carbohydrate intakes (g/day) based on dietary records and give patients individual targets for delta-reductions in carbohydrate intake from baseline, in our experience doing this is too time consuming. Our results indicated the amount of carbohydrate reduction necessary to achieve a certain decrease in HbA1c levels in each group. They will allow us to give clear and accurate goals for carbohydrate delta-reduction from baseline to individual patients. However, considering that it is not easy to assess quantities of carbohydrate intake at baseline, it would be more practical to start instruction by telling patients to eliminate carbohydrate-rich food once or twice daily, at breakfast and/or dinner, according to baseline HbA1c levels, without assessing carbohydrate intakes. If a patient could not achieve an individual optimal target HbA1c level after 3–6 months, we would modify the quantity of daily carbohydrate intake based on the current findings. To aid the instruction of patients in this regard, a list of foods giving their carbohydrate contents (e.g. 60 g carbohydrate in a bowl of rice and 30 g carbohydrate in a slice of bread) would be accurate enough. Despite the difficulty, accurate assessment of carbohydrate consumption at baseline and during the course of the dietary treatment would give patients more consistent carbohydrate and energy deficits.

Several reviews on LCDs have mentioned the 2 different ways of expressing carbohydrate restriction (i.e., g/day and %) [1,2]. The current study demonstrates that baseline HbA1c levels were correlated with carbohydrate intake (g/day) but not with %carbohydrate. Also, Δcarbohydrate (g/day) was very strongly (inversely) correlated with baseline carbohydrate intake (g/day), but weakly with Δ%carbohydrate, and ΔHbA1c was strongly correlated with Δcarbohydrate (g/day), but weakly with Δ%carbohydrate. Furthermore, g/day is more intuitionally acceptable to patients than% carbohydrate when instructing them. This suggests that expression of carbohydrate intake in g/day is superior to expressing it as %carbohydrate for the management of patients with type 2 diabetes given moderate LCDs.

The first limitation of our study is that the results could be partly due to changes in exercise amounts and medications. Indeed, the number of patients on anti-diabetic drugs decreased in the study period, especially in Group 2. Further studies on patients not taking anti-diabetic drugs will be required to resolve this issue. The second limitation is that we did not directly compare 3-graded moderate LCD with 2-graded moderate LCD. A direct comparison will be required to determine whether more detailed stratification of carbohydrate restriction by levels of baseline HbA1c can allow patients with lower HbA1c levels to avoid unnecessarily strict restriction of carbohydrate intake and still achieve sufficient glycemic control. The third limitation is that though the stricter carbohydrate restriction imposed on patients with HbA1c ≥ 9% achieved a considerable decrease in HbA1c levels, a similar result might have been achieved with less strict carbohydrate restriction. A study design in which patients are randomly assigned to 3-graded stratification (i.e., regardless of patient’s baseline HbA1c level) might provide useful findings in this regard. Although better glycemic control was achieved by a greater reduction in carbohydrate intake in this study, the long-term safety of LCDs has not been proved by interventional studies. In view of this, we believe it important to know the minimal carbohydrate restriction that is effective for glycemic control as well as the maximal carbohydrate restriction that is feasible for patients. At the same time, we should not hesitate to impose stricter carbohydrate restriction on patients when necessary.

In conclusion, the 3-graded stratification of carbohydrate restriction depending on patients’ baseline HbA1c levels achieved HbA1c levels of around 7.0% after 6 months despite great differences in baseline HbA1c levels and carbohydrate intake. We found that the greater the reduction in carbohydrate intake (g/day), the greater the decrease in HbA1c levels. We also demonstrated that the amount of carbohydrate reduction necessary to achieve a certain HbA1c decrease in each group. Our dietary strategy may provide patients with type 2 diabetes with optimal and practical objectives for carbohydrate restriction and prevent restriction from being unnecessarily strict.

## Competing interests

The authors declare that they have no competing interests.

## Authors’ contributions

HH and TS designed the study. HH and TS participated in data collection. HH, TS and KW performed the statistical analysis and all authors interpreted the data. HH and MK wrote the manuscript. All authors read and approved the final manuscript.
